# High resolution carotid black-blood 3T MR with parallel imaging and dedicated 4-channel surface coils

**DOI:** 10.1186/1532-429X-11-41

**Published:** 2009-10-27

**Authors:** Tobias Saam, Jose G Raya, Clemens C Cyran, Katja Bochmann, Georgios Meimarakis, Olaf Dietrich, Dirk A Clevert, Ute Frey, Chun Yuan, Thomas S Hatsukami, Abe Werf, Maximilian F Reiser, Konstantin Nikolaou

**Affiliations:** 1 Dept of Clinical Radiology, University of Munich, Grosshadern Campus, Munich, Germany; 2 Josef Lissner Laboratory for Biomedical Imaging, Dept of Clinical Radiology, University of Munich, Grosshadern Campus, Munich, Germany; 3 Dept of Surgery, University of Munich, Grosshadern Campus, Munich, Germany; 4 Dept of Radiology, University of Washington, Seattle, WA, USA; 5 VA Puget Sound Health Care System, Seattle Division, 1660 South Columbian Way, Seattle, WA 98108, USA; 6 Dept of Surgery, University of Washington, Seattle, WA, USA; 7 Machnet BV, TD Eelde, the Netherlands

## Abstract

**Background:**

Most of the carotid plaque MR studies have been performed using black-blood protocols at 1.5 T without parallel imaging techniques. The purpose of this study was to evaluate a multi-sequence, black-blood MR protocol using parallel imaging and a dedicated 4-channel surface coil for vessel wall imaging of the carotid arteries at 3 T.

**Materials and methods:**

14 healthy volunteers and 14 patients with intimal thickening as proven by duplex ultrasound had their carotid arteries imaged at 3 T using a multi-sequence protocol (time-of-flight MR angiography, pre-contrast T1w-, PDw- and T2w sequences in the volunteers, additional post-contrast T1w- and dynamic contrast enhanced sequences in patients). To assess intrascan reproducibility, 10 volunteers were scanned twice within 2 weeks.

**Results:**

Intrascan reproducibility for quantitative measurements of lumen, wall and outer wall areas was excellent with Intraclass Correlation Coefficients >0.98 and measurement errors of 1.5%, 4.5% and 1.9%, respectively. Patients had larger wall areas than volunteers in both common carotid and internal carotid arteries and smaller lumen areas in internal carotid arteries (p < 0.001). Positive correlations were found between wall area and cardiovascular risk factors such as age, hypertension, coronary heart disease and hypercholesterolemia (Spearman's r = 0.45-0.76, p < 0.05). No significant correlations were found between wall area and body mass index, gender, diabetes or a family history of cardiovascular disease.

**Conclusion:**

The findings of this study indicate that high resolution carotid black-blood 3 T MR with parallel imaging is a fast, reproducible and robust method to assess carotid atherosclerotic plaque in vivo and this method is ready to be used in clinical practice.

## Introduction

Complications of cardiovascular disease, including stroke, myocardial infarction, and sudden cardiac death, are the most common causes of death in the western world. The challenge for screening and diagnostic methods is to identify patients at high risk who have lesions that are vulnerable to thrombosis, so-called "vulnerable plaques", before the event occurs. Imaging methods have the potential to not only be used as a screening tool for the presence of atherosclerosis but also to help distinguish stable from vulnerable plaques and finally to distinguish between patients with low risk from those with high risk of cardiovascular complications[1].

Traditionally, atherosclerosis imaging was focused on the depiction of the arterial lumen[2]. However, atherosclerosis is a disease which begins in the arterial wall and hemodynamically relevant luminal narrowing potentially occurs rather late in the atherosclerotic disease process. Therefore, it is now commonly accepted[3,4] that knowledge of the luminal diameter is not sufficient to determine the vulnerability of an atherosclerotic lesion, and a number of major and minor criteria have been proposed for the assessment of a vulnerable plaque. These plaque features were based on studies of coronary arteries and included thin caps with large lipid/necrotic core, active inflammation, fissured plaque, stenosis >90%, endothelial denudation with or without superficial platelet aggregation and fibrin deposition, endothelial dysfunction, calcified nodules, intraplaque hemorrhage, glistening yellow plaques (on angioscopy), and outward remodeling. Hence, the ideal atherosclerosis imaging method should not only be able to quantify the degree of luminal stenosis but also to identify these key features defining a vulnerable plaque.

Although several imaging modalities, such as intravascular ultrasound (IVUS)[5] or computed tomography[6], have been in use to assess atherosclerotic plaques, magnetic resonance (MR) provides the unique potential to identify most of the key features of the vulnerable carotid plaque[1]. MR is non-invasive, does not involve ionizing radiation, enables the visualization of the vessel lumen and wall[7] and can be repeated serially to track progression or regression[8]. Furthermore, the excellent soft tissue contrast provided by MR enables the evaluation of compositional and morphological features of atherosclerotic plaques[1].

Recent MR studies. [8-12] have shown that this method could become a useful clinical tool to assess the carotid atherosclerotic plaque. However, many of these carotid plaque MR studies. [8-10] have been performed using black-blood protocols at 1.5 T without parallel imaging techniques. Therefore, disadvantages of previous carotid MR studies at 1.5 T were long scan times of up to 45 minutes and a relatively high number of excluded subjects due to insufficient image quality in 5-33% of the subjects[13]. Furthermore, the spatial resolution of 0.6 × 0.6 mm typically used for 1.5 T MR studies might not be able to identify very small features of the atherosclerotic plaque, such as the fibrous cap.

Recently, clinical MR at 3 T, which can potentially increase the signal obtained during imaging by a factor of 2, has become widely available and several carotid black-blood 3 T MR studies [11,12,14,15] have shown that the added signal can be used to either decrease scan time or to reduce spatial resolution or a combination of both. However, these carotid MR studies [11,12,14,15] did not use parallel imaging techniques. Parallel imaging techniques are being used increasingly in clinical practice [16] and have been used recently for aortic atherosclerosis imaging using a sensitivity encoding (SENSE) algorithm [17]. Briefly, parallel acquisition techniques combine the signals of several coil elements in a phased array to reconstruct the image, the chief objective being either to improve the signal-to-noise ratio or to accelerate acquisition and reduce scan time. Shorter scan times could potentially reduce motion artefacts caused by respiration and swallowing.

To further improve carotid black-blood imaging and to facilitate its usefulness in routine clinical practice we propose a multi-sequence carotid black-blood protocol using parallel imaging and a dedicated 4-channel surface coil on a 3 T MR scanner, to reduce acquisition time per slice by factor 2, minimize dropouts and optimize overall image quality. To evaluate the clinical usefulness of our protocol we evaluated the reproducibility of quantitative plaque measurements and we compared compositional and morphological plaque features between healthy volunteers and subjects with carotid atherosclerotic disease.

## Materials and methods

### Patients, MR scanner and carotid coil

The study was approved by the institutional review board and all subjects gave written informed consent. 14 healthy volunteers and 14 patients with intimal thickening as proven by duplex ultrasound had their carotid arteries (both sides) imaged at 3.0-T (Magnetom Verio, Siemens Healthcare, Erlangen, Germany). To improve signal-to-noise performance and optimize spatial resolution, a dedicated four-channel surface coil (Machnet, Eelde, Netherlands) for bilateral carotid scans was used. The flexible coil design allows to combining this coil with all other available coils such as head and/or neck coils. To evaluate the intra-scan reproducibility of this method, 10 volunteers were scanned twice within two weeks. Thus, in total 38 MR exams were performed.

### MR Protocol

All subjects were imaged using a multi-sequence protocol without ECG gating (see Table 1, time-of-flight MR angiography (TOF), axial pre-contrast T1-, PD- and T2- weighted (T1w, PDw, T2w) in the volunteers and additional axial post-contrast T1w and dynamic contrast-enhanced sequences (DCE) in patients; best in-plane resolution 0.5 × 0.5 mm^2^). Parallel imaging based on the generalized autocalibrating partially parallel acquisition (GRAPPA) algorithm[18] was used for all sequences with a parallel acquisition technique (PAT) acceleration factor of 2 for TOF, T1w, PDw and T2w images and a PAT factor of 4 for DCE images. Imaging time for TOF, T1w, PDw, T2w and DCE images were 4:11, 4:38, 2:08, 2:08 and 5:00 minutes, respectively, resulting in a total scan time of 13:05 minutes in volunteers and 22:43 minutes in patients. DCE was performed for five minutes during the intravenous injection of 0.1 mmol/kg (0.1 ml/kg) Gadolinium-DTPA-BMA (Gadobutrol, Bayer Schering, Leverkusen, Germany) at a rate of 3 mL/s. Two slices at different locations were acquired every 1.8 s, resulting in a total of 167 acquisitions per slice. Post-contrast T1w imaging was performed approximately 5 minutes after intravenous injection of the contrast agent. Fat suppression was used for pre- and post-contrast T1w, PDw, and T2w images to reduce signals from subcutaneous and perivascular fat. Each scan covered 30 mm (2 mm slice thickness × 15 matched images across the 5 sequences). This coverage is usually sufficient to image the complete carotid atherosclerotic plaque[19].

**Table 1:** MR Protocol

	**T1W**	**PDW**	**T2W**	**TOF**	DCE*
**Sequence**	2D-TSE	2D-TSE	2D-TSE	3D-GRE	2D-SR-SGRE

**ECG gating**	None	None	None	None	None

**Fat Suppression**	Yes	Yes	Yes	Yes	No

**TR [ms]**	800	3000	3000	21	307

**TE [ms]**	12	13	65	3.96	1.72

**PAT factor**	2	2	2	2	4

**ETL**	11	13	13	n.a.	n.a.

**Flip Angle [°]**	180	180	180	25	15

**Averages**	2	2	2	1	3

**FOV [mm^2^]**	160 × 120	160 × 120	160 × 120	160 × 120	160 × 130

**Matrix**	240 × 320	240 × 320	240 × 320	240 × 320	256 × 208

**Number of Slices**	15	21	21	52	2

**Slice thickness [mm]**	2	2	2	1	3.5

**Pixel size [mm^2^]** **(Interpolated)**	0.5 × 0.5 (0.25 × 0.25)	0.5 × 0.5 (0.25 × 0.25)	0.5 × 0.5 (0.25 × 0.25)	0.5 × 0.5 (0.25 × 0.25)	0.625 × 0.625

**Scan Time per slice (Sequence)** **[minutes]**	0:19 (4:38)	0:06 (2:08)	0:06 (2:08)	0:05 (4:11)	0:02 (5:13)

**Flow Suppresion**	DIR	Inflow Suppression (Arteries and Veins)	Inflow Suppression (Arteries and Veins)	Inflow Suppression (Veins)	None

DCE = Dynamic Contrast Enhanced Sequences; *TI = 159 ms; D = Dimensional; TSE = Turbo Spin Echo; SR-SGRE = Saturation-Recovery Spoiled Gradient Echo, PAT = Parallel Acquisition Technique; ETL = Echo Train Length; DIR = Double Inversion Recovery

### Intra-scan and Inter-scan Matching

**Figure 1:**
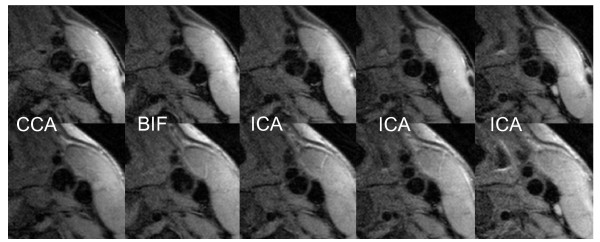
**T1- weighted images with average image quality (ImQ = 3) of the left carotid artery (lumen = asterisk) of the MR scan of a volunteer who was scanned twice within two weeks**. Images are well matched and appear almost identical (1st column: CCA = common carotid artery, 2nd column: BIF = bifurcation, 3^rd^, 4^th ^and 5^th ^column: ICA = internal carotid artery).

Reviewers were blinded to subject, time point, and clinical information. Previous studies [20,21] have shown that information from multiple sequences are needed for accurate identification of plaque tissue components. Therefore, cross-sectional locations from all the five contrast weightings of each carotid artery from a single MR examination were matched relative to the bifurcation. The bifurcation was assigned to the location 2 mm below the location, where the lumen of the common carotid artery separates into the two lumen of the internal and external carotid artery. In order to insure a similar coverage of the plaque for the reproducibility study, only image locations that could be matched across the two scans were reviewed. Figure 1 illustrates the inter-scan matching across the two exam time points.

### Image Review

Each scan was read by two reviewers with extensive experience in carotid plaque imaging who reached consensus decision. An image-quality rating (4-point scale, 1 = non-diagnostic, 2 = poor, 3 = good, 4 = excellent) was assigned to all MR images[22] before the review. Arteries in which the bifurcation was not covered were excluded from the review (n = 3).

Area measurements of the lumen, outer wall, and tissue components were obtained using a custom-designed image analysis tool CASCADE (University of Washington, Seattle, US)[23]. The "total vessel area" included the lumen and wall areas. The wall area for each location was calculated as the difference between the total vessel and lumen areas. The normalized wall index (NWI) was calculated by dividing the wall area by the total vessel area. Tissue components (lipid-rich necrotic core, calcification, hemorrhage) were identified based on previously published criteria[20]. All signal intensities (SI) given are relative to the normal carotid artery wall. Post-contrast T1w images were used to manually outline the plaque components because it has been shown that they are best suited to quantify the lipid rich/necrotic core (LR/NC)[24,25]. The LR/NC is generally located in the bulk of the plaque and has varied SI depending on the amount and age of potential hemorrhage present. LR/NC's without hemorrhage are isointense on all four weightings and can be best depicted on post-contrast T1w images in which they show no contrast enhancement compared to the surrounding enhancing fibrous tissue and the surrounding muscular layer of the arterial wall. LR/NC's with hemorrhage are hyperintense on TOF and T1w images. SI on PDw and T2w images depends on the type of hemorrhage within the LR/NC[21,26]. Briefly, type I hemorrhage is iso- to hypointense on PDw- and T2w images and type II hemorrhage has high signal intensity on all 4 weightings. Calcification was identified as a hypointense signal on all 4 weightings[20].

### Data Analysis

Measurement errors for mean area measurements of lumen, wall and total vessel area were calculated as 100%* √ [within-patient variance]/Mean (all measurements). The Intraclass Correlation Coefficient (ICC) was calculated to measure the level of agreement between two repeated measurements within subjects in comparison to the variation in the measurement across subjects. An ICC close to 1.0 indicates that measurement error is small relative to the range of values encountered. For statistical comparison of the patients and the volunteers, the Mann-Whitney Test with correction for ties was used for variables describing frequencies of plaque features, which had skewed distribution. For continous variables, Student's unpaired t-test with equal or unequal variances was used. To compare clinical demographics between both groups, Fisher's Exact Test was used for categorical variables and the unpaired t-test was used for continuous variables. Spearman's correlation coefficient was used to correlate age and clinical risk factors with quantitative plaque measurements. Multiple linear regression analysis was used to accommodate for the differences in risk factors. Analyses were carried out in SPSS for windows (SPSS 10.0, SPSS Inc., Chicago, IL, USA).

## Results

### MR Scans, Image Quality and Mean Coverage

37 out of 38 scans (97.4%) had a sufficient image quality (image quality ≥ 2), with an average image quality rating of 3.3 for volunteers and 3.3 for patients (p = n.s.). The non-diagnostic scan was of a patient with a very short neck, impeding adequate coil positioning. Three arteries of volunteers were excluded because the bifurcation was covered only on one side. In total, 71 arteries were reviewed. Mean coverage per artery was 2.2 ± 0.6 cm. In total 3905 MR images were reviewed.

### Descriptive Characteristics (Table 2)

**Table 2:** Demographics and Plaque Characteristics

	**Volunteers (n = 14)** **Mean ± SD or %**	**Patients (n = 13)** **Mean ± SD or %**	p-value*
***I. Demographics and Risk Factors***			
Age [years]	32.6 ± 11.7	70.5 ± 8.3	P < .001
Body Mass Index	23.8 ± 2.3	24.1 ± 1.9	n.s.
Male Sex [%]	50%	69.2%	n.s.
Coronary Artery Disease [%]	0%	46.2%	P < .05
Hypertension [%]	0%	61.5%	P < .05
Hypercholesterolemia [%]	7.1%	69.2%	P < .05
Diabetes [%]	0%	30.8%	P < .05
Family History of CVD [%]	21.4%	23.1%	n.s.
Active Smoker [%]	57.1%	15.4%	P < .05
***II. Plaque Characteristics***			
Mean Lumen Area CCA [mm^2^]	41.92 ± 17.55	41.17 ± 14.36	n.s.
Mean Lumen Area ICA [mm^2^]	34.91 ± 13.41	16.63 ± 7.26	P < .05
Mean Wall Area CCA [mm^2^]	17.47 ± 8.05	43.80 ± 15.38	P < .001
Mean Wall Area ICA [mm^2^]	14.77 ± 6.80	33.80 ± 14.07	P < .05
Mean Total Vessel Area CCA [mm^2^]	59.39 ± 23.95	84.97 ± 28.44	P < .05
Mean Total Vessel Area ICA [mm^2^]	49.69 ± 18.22	50.44 ± 16.62	n.s.
Mean NWI CCA	0.30 ± 0.06	0.50 ± 0.04	P < .001
Mean NWI ICA	0.30 ± 0.08	0.66 ± 0.12	P < .001
Lipid-Rich Necrotic Core** [%]	3.7%	76.9%	P < .05
Calcification** [%]	0%	73.1%	P < .05
Hemorrhage** [%]	0%	46.2%	P < .05

*Fisher's Exact Test for categorical variables and unpaired t-test for continuous variables, CVD = Cardiovascular Disease; CCA = Common Carotid Artery; NWI = Normalized Wall Index; ** Prevalence given as the percentage of analyzed arteries showing one feature

Patient and volunteers baseline clinical information are given in table 2. Patients were significantly older than volunteers and were more likely to have hypertension, hypercholesterolemia, diabetes, coronary heart disease (all p-values < 0.05). No significant differences between patients and volunteers were found for body mass index, gender and family history of cardiovascular disease. Volunteers were more likely than patients to smoke (p < .05).

### Plaque Characteristics (Table 2, Figure 2, 3, 4)

**Figure 2:**
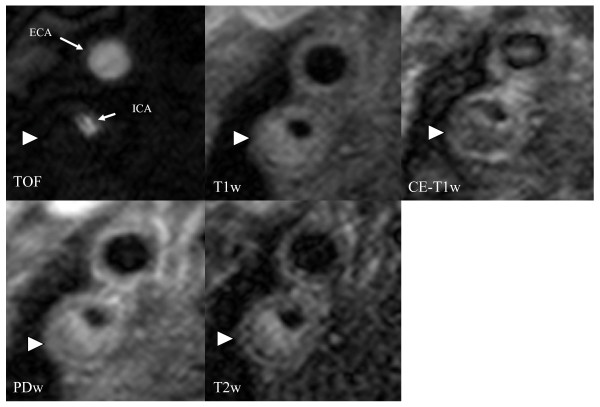
**MR images of a 74 year old asymptomatic patient with a 80% carotid stenosis in his right internal carotid artery (ICA; ECA = external carotid artery)**. Cross- sectional MR images show a large eccentric plaque in the right ICA with a large necrotic core without intraplaque hemorrhage (arrow), which is covered by a thick layer of dense and loos fibrous tissue, which can be depicted on PD- and T2w- images as a hyperintense region near the lumen surface which shows moderate contrast enhancement on the CE-T1w images. The plaque is classified as an American Heart Association Lesion Type IV/V with a thick fibrous cap.

**Figure 3:**
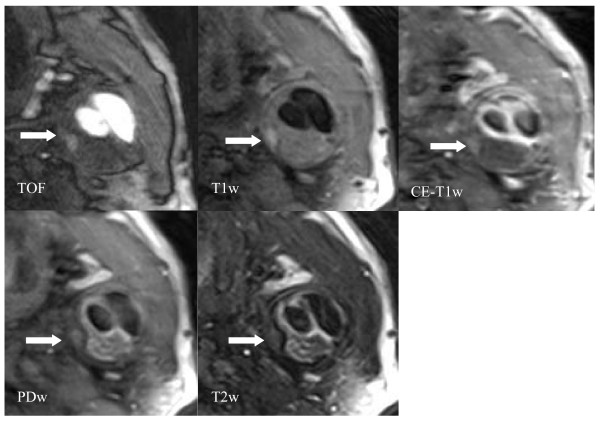
**Axial images show a large eccentric plaque in the left common carotid artery of a 53-year old patient**. The arrow points to an area of high signal intensity on TOF and T1w images which indicates intraplaque hemorrhage. Post-contrast T1w images allow the delineation of the lipid-rich necrotic core, which does not show any contrast uptake. The lipid-rich necrotic core is covered by a layer of fibrous tissue, which is hyperintense on the PDw and T2w images, consistent with an intact fibrous cap. The plaque is classified as a complicated American Heart Association Lesion Type VI.

**Figure 4:**
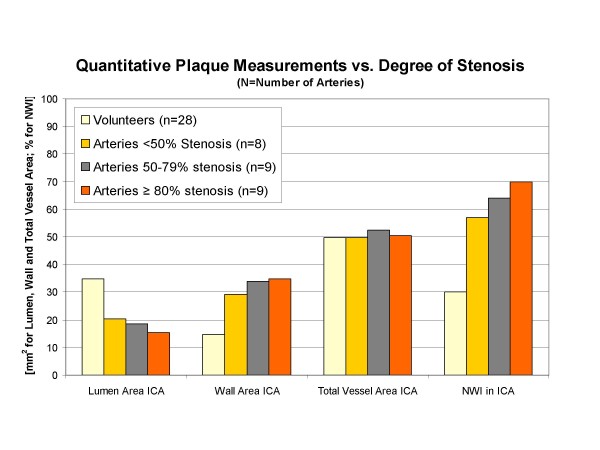
**Differences between arteries with varying degrees of stenosis according to duplex ultrasonography for quantitative carotid atherosclerotic plaque measurements in the internal carotid artery (ICA; NWI = Normalized Wall Index)**.

Plaque characteristics of volunteers and patients are given in table 2. Mean wall area and mean NWI in both common (CCA) and internal carotid artery (ICA) were significantly larger in patients than in volunteers. No significant differences between patients and volunteers were found for mean lumen area in the CCA and for the mean total vessel area in the ICA. Mean lumen area was significantly smaller in the ICA and mean total vessel area was significantly larger in the CCA in patients compared to volunteers. One 57-year old volunteer had a lipid-rich necrotic core in the right CCA, all other volunteers had normal or near-normal carotid arteries. Lipid-rich necrotic cores were found in 77% of the arteries of the patients, calcification in 73% and hemorrhage in 46%. Figure 2 demonstrates a lesion in the right internal carotid artery with a lipid-rich necrotic core which is covered by an intact and thick fibrous cap. Figure 3 demonstrates a lesion in the left CCA with a large necrotic core, intraplaque hemorrhage and an arterial dissection.

Figure 4 demonstrates results of quantitative plaque measurements in the ICA according to the degree of stenosis as measured by duplex sonography. Mean lumen area decreases with higher degrees of stenosis while mean wall area and mean NWI increases substantially. No trend was seen between total vessel area and degree of stenosis.

### Intraclass Correlation Coefficient (Table 3)

**Table 3:** Protocol Parameters and Reproducibility of Quantitative Measurements on 3 T MR: Comparison with previous 1.5 T MR studies

	**3.0-T**	**1.5 T** [20,22,30]	**Difference**
**Interpolated Pixel Size [mm^2^]**	0.25 mm × 0.25 mm = 0.0625 mm^2^	0.3 mm × 0.3 mm = 0.09 mm^2^	-31%

**Scan Range [mm]**	30	24	+ 25%

**Total Scan Time [minutes]**	13:05 Protocol CM^-^ 22:43 Protocol CM^+^	19:30 CM^-^ 31:30 CM^+^	-33% -28%

**Scan Time per Slice [seconds]**			
**T1w**	18.5	35.0	-47%
**PD/T2w**	6.1	15.0	-59%
**TOF**	4.8	9.6	-50%

**Non-Diagnostic Exams [%]**	1/38 [2.6%]	27/252 [10.7%]	

**PAT factor**	2 DCE = 4	1	+100%

**Coefficient of Variation**			
**a.) Lumen Area**	1.5%	4.3%	
**b.) Wall Area**	4.5%	5.8%	
**c.) Total Vessel Area**	1.9%	3.3%	
**d.) NWI**	3.0%	n.a.	

**ICC (95% CI)**			
**a.) Lumen Area**	1.00 (0.99-1.00)	0.99 (0.98-1.00)	
**b.) Wall Area**	0.98 (0.93-1.00)	0.97 (0.95-0.99)	
**c.) Total Vessel Area**	1.00 (0.99-1.00)	0.98 (0.97-1.00)	
**d.) NWI**	0.88 (0.60-0.97)	n.a.	

Protocol CM^- ^= no contrast media used, time for TOF, PDw, T2w and T1w images; CM^+ ^= same as CM^-^, + contrast-enhanced T1w and dynamic contrast enhanced sequences added; PAT = Parallel Acquisition Technique; DCE = Dynamic Contrast Enhanced Sequences; ICC = Intra Class Correlation Coefficient; NWI = Normalized Wall Index; CI = Confidence Interval

The ICC showed excellent intra-scan agreement for all measurements, indicating that the variation in the measurements across subjects was much higher than the variation of repeated measurements within subjects. The ICC for mean lumen, wall and total vessel area measurements ranged from 0.98-1.00 (see table 3). The ICC for the mean NWI was 0.88.

### Measurement error

Intra-scan measurement errors for lumen, wall and total vessel areas were 1.5%, 4.5% and 1.9%, respectively (see table 3). Intra-scan measurement error for NWI was 3.0% (see table 3).

### Correlation of Quantitative Plaque Measurements with Age, Gender and Cardiovascular Risk Factors

Table 4 demonstrates the correlation of baseline parameters with quantitative measurements of carotid atherosclerotic plaque burden in the CCA and ICA. Mean wall area and NWI showed positive correlations in CCA and/or ICA with age and all cardiovascular risk factors except family history of cardiovascular disease. In general, correlation coefficients of NWI with age and risk factors tended to be slightly larger than correlation coefficients of wall area with age and risk factors. Smoking was negatively correlated with mean wall area and mean NWI in the CCA. However, prevalence of smoking was significantly higher in the volunteers and the correlation between NWI, mean area and smoking did not remain statistically significant when we used a multiple regression analysis to accommodate for differences in age and risk factors. Mean lumen area was negatively correlated with age and hypercholesterolemia in the ICA and positively correlated with male gender in the CCA. No other significant correlations were found for mean lumen area. Mean total vessel area showed a significant positive correlation in the CCA with age and coronary heart disease. No other significant correlations were found for mean total vessel area.

**Table 4:** Correlation of Baseline Parameters with Quantitative Measurements of Carotid Atherosclerotic Plaque Burden in the Common Carotid Artery/Internal Carotid Artery

***Spearman's R*** ***CCA/ICA***	***Mean Lumen Area***	***Mean Wall Area***	***Mean Total Vessel Area***	***Mean NWI***
Age	n.s./-0.57*	0.76**/0.57*	0.40*/n.s.	0.82**/0.76*

Body Mass Index	n.s./n.s.	n.s./n.s.	n.s./n.s.	n.s./n.s.

Male Gender	0.59*/n.s.	n.s./n.s.	0.61*/n.s.	n.s./n.s.

Diabetes	n.s./n.s.	n.s./0.56*	n.s./n.s.	n.s./0.56*

Hypercholesterolemia	n.s./-0.59*	0.61*/n.s.	n.s./n.s.	0.68**/0.49*

Hypertension	n.s./n.s.	0.53*/0.57*	n.s./n.s.	0.64*/0.60*

Family History of CVD	n.s./n.s.	n.s./n.s.	n.s./n.s.	n.s./n.s.

Coronary Heart Disease	n.s./n.s.	0.45*/n.s.	0.41*/n.s.	n.s./0.47*

Active Smoker	n.s./n.s.	-0.43*/n.s.	n.s./n.s.	-0.47*/n.s.

CCA = Common Carotid Artery; ICA = Internal Carotid Artery, NWI = Normalized Wall Index; *p < 0.05; **p < 0.001; CVD = Cardiovascular Disease

## Discussion

The results of this study demonstrate that high-resolution 3 T carotid MR with parallel imaging has a high reproducibility for quantitative measurements of lumen area, wall area, total vessel area and NWI. With our standardized protocol, implementing parallel imaging techniques and dedicated surface coils, we achieved sufficient image quality and coverage to perform quantitative measurements in 37 out of 38 scans and good intra-scan reproducibility was obtained across the two different time points in the 10 volunteers which were measured twice, optimizing the co-registration by using the bifurcation as a landmark (figure 1). Furthermore, this study was able to show highly significant differences of quantitative and qualitative plaque measurements between the 14 volunteers and the 13 patients, demonstrating that MR is able to detect significant differences between populations, even in small patient cohorts.

Similarly to previous studies on 1.5 T [22,27], and 3 T MR systems [11,15,28] this study showed excellent correlation in the 10 volunteers for the baseline measurements compared to the second time point for all measured variables. This indicates that the normal variation between subjects of any of the measurements was much greater than the variation within the repeated measurements and expresses almost perfect agreement on repeated measurements (perfect agreement = 1.00). In contrast to previous studies [11,15,22,27,28] this was achieved by using parallel imaging techniques, which accelerated the image acquisition per slice by a factor of 2 for TOF, PDw, T2w and T1w images (see table 3),

The measurement error or modified coefficient of variation ranged from 1.5-4.5% for mean lumen area, mean wall area, mean total vessel area and NWI and was slightly lower than previously reported in 1.5 T MR studies (see table 3 for comparison of 1.5 T and 3 T MR studies) in single [29] and multi-center trials [22] (5-6.8% for mean wall area measurements). Possible reasons for the lower measurement error reported in the current study might be due to the following reasons: i.) improvement of the spatial resolution which resulted in a 31% smaller voxel size compared to previous 1.5 T MR studies, ii.) use of parallel imaging techniques which resulted in a decrease of scan time and thus less time for possible patient movement and iii.) signal gains associated with the higher field strength which might have resulted in an improved lumen and wall boundary detection.

Due to the low variability of quantitative measurements, MR is able to detect differences between groups even in small study populations. This study was able to show significant differences in the CCA and/or ICA between 13 patients and 14 volunteers for all quantitative measurements and for the prevalence of features of advanced atherosclerotic disease, such as lipid-rich necrotic core, calcification or hemorrhage. As expected, patients had larger mean wall areas and mean NWI's in both CCA and ICA, which confirms the larger atherosclerotic plaque burden in patients. Total vessel area in the CCA was significantly larger in patients compared to volunteers, which indicates that the CCA in the patients had undergone outward remodeling, while mean lumen area in the CCA was preserved

This study found positive correlations with mean wall area and NWI in the CCA and/or ICA with age and all cardiovascular risk factors except family history of cardiovascular disease. This finding indicates that both NWI and mean wall area might be useful to assess the atherosclerotic disease burden. On the other side, mean lumen area failed to show significant correlations with cardiovascular risk factors except with hypercholesterolemia in the ICA, suggesting that assessment of mean lumen area is a less sensitive marker of the atherosclerotic disease burden than NWI and mean wall area. Interestingly, correlation coefficients of NWI with age and risk factors tended to be slightly larger than correlation coefficients of wall area with age and risk factors, suggesting that NWI might be the most sensitive measure to evaluate the severity of the atherosclerotic disease process.

In contrast to previous 1.5 T and 3.0-T MR studies [11,15,22,27,28], the 3 T MR study protocol presented here used parallel imaging techniques to image the bilateral carotid arteries. The technique was based on the generalized autocalibrating partially parallel acquisition (GRAPPA) algorithm [18] with a PAT factor of 2 for the TOF, T1w, T2w and PDw images and a PAT factor of 4 for the dynamic contrast enhanced sequences. This was associated with a substantially shorter scan time per slice which decreased between 47% and 59% for T1w, PDw/T2w and TOF images (see table 3). In order to ensure full coverage of the carotid atherosclerotic plaque bilaterally, the scan range was expanded from 24 to 30 mm. Thus, despite the increased coverage this protocol resulted in a total decrease of the scan time of 33% for non-contrast enhanced exams and 29% for contrast-enhanced exams.

A recent 3 T carotid MR study[28] has shown significant improvement in SNR, CNR, and image quality for high- resolution black-blood imaging of carotid arteries at 3 T compared to 1.5 T with a 1.5-fold SNR gain for T1-weighted images and a 1.7/1.8-fold gain for PD-/T2-weighted images. Another study [15] showed signal gains for carotid artery wall SNR at 3.0 T relative to 1.5 T of 223% and wall-lumen CNR of 255% in all acquisitions (P < 0.025). The signal gain associated with the higher field strength and the shorter scan time due to the parallel imaging technique was used to establish a clinical MR protocol which was faster, had a higher longitudinal coverage and a better spatial resolution than protocols previously used on 1.5 T MR scanners (see table 3) [22,27]. This study achieved diagnostic image quality in 37 out of 38 scans, demonstrating that this protocol can be successfully used in a clinical setting. This drop-out rate is substantially lower than the drop-out rates reported in recent 1.5 T MR studies[20,22,30], in which 27 out of 252 (10.7%) exams were excluded due to insufficient image quality. It is also substantially lower than the drop-out rate reported in a recent multi-center MR study [13] in which 160 patients with greater than 50% carotid artery stenosis were recruited at six centers for prospective imaging of the carotid arteries at baseline and 1 year later by using high-spatial-resolution, 1.5 T MR imaging. All 160 patients completed both baseline and follow-up studies. Of these studies, only 67.5% were deemed to have image quality that was acceptable for quantitative analysis. The causes of rejection were motion (46%), deep location of the carotid artery (22%), low bifurcation of the carotid artery (13%), and "other" (19%). One of the reasons of the lower number of excluded exams in our study compared to previous 1.5 T MR studies might be the signal gains associated with the higher field strength in combination with the shorter scan time due to the Parallel Imaging technique, which is easier to tolerate for the patients and might therefore result in less motion artefacts and artefacts due to patient swallowing.

Recent 3 T carotid MR studies [14,15] used coil designs in which the carotid coil could not be combined with other coils, such as head or neck coils. In contrast, this study used a dedicated 4-channel surface coil, which can be easily combined with all other commercially available coils for the 3 T imager used in this study. This is a substantial advantage over other coil designs as with this approach, an MR examination of the carotid arterial wall and a brain MR and/or an angiography of the supraaortal vessels can be performed without a coil change or patient repositioning.

## Conclusion

The results of this study demonstrate that imaging of the carotid arterial wall on a 3 T imager with dedicated surface coils and parallel imaging techniques has a number of advantages, including shorter overall imaging time, higher spatial resolution, larger volume coverage, and improved overall image quality with a lower number of excluded exams due to insufficient image quality as compared to 1.5 T carotid plaque imaging. Also, 3 T carotid MR with parallel imaging has a lower variability for quantitative measurements of lumen area, wall area, total vessel area and NWI compared to previous 1.5 T studies. Significant differences between quantitative and qualitative plaque measurements were found between patients and volunteers, and highly significant correlations were found between mean wall areas and mean NWIs with age and all cardiovascular risk factors, except family history of cardiovascular disease. These results demonstrate that in vivo MR is able to detect significant differences between groups even in small patient cohorts and provides further evidence that carotid plaque composition and morphology is correlated with atherosclerotic risk factors. In summary, carotid black-blood 3 T MR with parallel imaging is a fast, reproducible and robust method to assess carotid atherosclerotic plaque *in vivo *and this method is ready to be used in clinical practice.

## Declaration of competing interests

The authors declare that they have no competing interests.

## Authors' contributions

TS, JGR, CCC, KB, GM, OD, DAC, UF, CY, TSH, AW, MFR and KN were involved in the study concept/study design. TS, KB and KN were involved in data analysis/interpretation.

TS, JGR, CCC, KB, GM, OD, DAC, UF, CY, TSH, AW, MFR and KN were involved in manuscript preparation and editing. TS, JGR, CCC, KB, GM, OD, DAC, UF, CY, TSH, AW, MFR and KN gave final approval of the submitted manuscript.
